# A Microclip Peripheral Nerve Interface (μcPNI) for Bioelectronic Interfacing with Small Nerves

**DOI:** 10.1002/advs.202102945

**Published:** 2021-11-26

**Authors:** Cami C. Rowan, Oliver Graudejus, Timothy M. Otchy

**Affiliations:** ^1^ BMSEED LLC Phoenix AZ 85034 USA; ^2^ School of Molecular Sciences Arizona State University Tempe AZ 85281 USA; ^3^ Department of Biology Boston University Boston MA 02215 USA; ^4^ Neurophotonics Center Boston University Boston MA 02215 USA; ^5^ Center for Systems Neuroscience Boston University Boston MA 02215 USA

**Keywords:** 3D printing, bioelectronic medicine, peripheral nerve interfaces, stretchable microelectrode arrays

## Abstract

Peripheral nerves carry sensory (afferent) and motor (efferent) signals between the central nervous system and other parts of the body. The peripheral nervous system (PNS) is therefore rich in targets for therapeutic neuromodulation, bioelectronic medicine, and neuroprosthetics. Peripheral nerve interfaces (PNIs) generally suffer from a tradeoff between selectivity and invasiveness. This work describes the fabrication, evaluation, and chronic implantation in zebra finches of a novel PNI that breaks this tradeoff by interfacing with small nerves. This PNI integrates a soft, stretchable microelectrode array with a 2‐photon 3D printed microclip (μcPNI). The advantages of this μcPNI compared to other designs are: a) increased spatial resolution due to bi‐layer wiring of the electrode leads, b) reduced mismatch in biomechanical properties with the nerve, c) reduced disturbance to the host tissue due to the small size, d) elimination of sutures or adhesives, e) high circumferential contact with small nerves, f) functionality under considerable strain, and g) graded neuromodulation in a low‐threshold stimulation regime. Results demonstrate that the μcPNIs are electromechanically robust, and are capable of reliably recording and stimulating neural activity in vivo in small nerves. The μcPNI may also inform the development of new optical, thermal, ultrasonic, or chemical PNIs as well.

## Graphical abstract

A peripheral nerve interface (PNI) for small nerves (100–200 µm) is developed to minimize the non‐specific or off‐target effects of neuromodulation. Soft, stretchable electrodes are integrated with a 2‐photon 3D printed microclip for an electromechanically robust yet compliant PNI. In vivo recording and stimulation inform new opportunities for therapeutic neuromodulation, bioelectronic medicine, and neuroprosthetics.

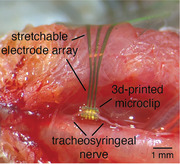

## 1 Introduction

Recording from and stimulating peripheral nerves is an area of increasing research interest and clinical importance, especially for restoring control of paralyzed muscles,^[^ ^1^ ^]^ dexterous command of advanced bionic limbs,^[^ ^2^ ^]^ and the therapeutic modulation of signaling in the peripheral nervous system (PNS) to alleviate pathological inflammation, pain, and other chronic disorders.^[^ ^3^, ^4^, ^5^, ^6^ ^]^ FDA‐approved clinical applications of peripheral nerve interfaces (PNIs) are largely limited to Vagus Nerve Stimulation (VNS) and Sacral Nerve Stimulation (SNS).^[^ ^7^ ^]^ The vagus nerve and the sacral nerve are both large diameter nerves (up to 4.8^[^ ^8^ ^]^ and 1.4 mm,^[^ ^9^ ^]^ respectively) carrying thousands of individual nerve fibers,^[^ ^9^ ^]^ thus the potential for unintended off‐target neuromodulatory effects is high.^[^ ^10^ ^]^ To minimize these unintended and off‐target effects, new PNIs are needed that 1) can chronically attach to small distal branches of the vagus and sacral nerves (<200 µm diameter), 2) establish high‐resolution recording and stimulation for precise targeting and modulation of signals within the terminal branches of the PNS, and 3) elicit desired modulatory effects without altering nerve health or other functionalities.^[^ ^11^, ^12^ ^]^

Peripheral nerves are heterogeneous viscoelastic structures, with elastic and shear moduli in the 5–500 kPa range, that exist in a biomechanically dynamic environment,^[^ ^13^, ^14^ ^]^ and accommodate body movement and local tissue strain through a combination of deformation and displacement.^[^ ^15^, ^16^, ^17^, ^18^, ^19^ ^]^ In contrast, most PNIs—even those of flexible thin‐film thermoplastics like polyimide (PI)^[^ ^20^, ^21^, ^22^, ^23^ ^]^ and Parylene‐C^[^ ^24^, ^25^, ^26^ ^]^—present high elastic moduli in the gigapascal range, and are thus rigid compared to body tissues.^[^ ^27^, ^28^ ^]^ Such PNIs that fail to accommodate the biomechanical properties of host tissues are unlikely to maintain stable recording and stimulation characteristics over time.

Conventional PNI technologies include cuff electrodes that envelop the nerve,^[^ ^29^, ^30^, ^31^, ^32^, ^33^, ^34^, ^35^, ^36^, ^37^ ^]^ sieve electrodes that provide mechanical guidance for regenerating nerves,^[^ ^38^, ^39^, ^40^, ^41^, ^42^, ^43^, ^44^ ^]^ stiff penetrating arrays,^[^ ^45^, ^46^ ^]^ and flexible arrays designed to be inserted longitudinally^[^ ^23^, ^38^, ^39^, ^41^, ^42^, ^43^, ^44^, ^45^, ^46^, ^47^ ^]^ or transverse to the direction of the fibers^[^ ^48^, ^49^ ^]^ (**Table** **1**). While there are distinct advantages to each of these approaches, none of them sufficiently addresses the biomechanical and environmental challenges required for long‐term reliable stimulation and recording.^[^ ^50^, ^51^ ^]^ Moreover, existing PNI technologies have a significant tradeoff between invasiveness (leading to nerve damage) and selectivity/sensitivity (a result of close proximity to the nerve fibers). For example, cuff electrodes are the least invasive, but are stiff and bulky (in comparison to body tissues) and suffer from limited selectivity for stimulation and a lack of recording sensitivity due to poor mechanical compliance with the nerve tissue and a comparatively large distance from the nerve fibers. Sieve electrodes are the most invasive because they require the nerve to be cut and regenerated through the sieve, with an unavoidable if transient damage phase and low probability of a recording site being close to the regrown nerve fibers.^[^ ^52^, ^53^ ^]^ Designs with penetrating probes have not yet demonstrated long‐term stability, have shown substantial scar‐tissue deposition within the nerve, and are prone to cause trauma in the nerve during implant.^[^ ^50^ ^]^ The Utah Slanted Electrode Array (USEA), for example, comprises a high‐density array of silicon shanks (Young's modulus >100 GPa)^[^ ^54^ ^]^ that penetrate the nerve to interface closely with the nerve fibers. However, the USEA exhibits a large mismatch in mechanical properties with the nerve which causes significant and irrecoverable^[^ ^55^ ^]^ nerve damage as well as scar tissue formation around the electrodes, limiting their effectiveness and usable lifetime. A chronically implantable PNI with high‐biomechanical compliance with host tissues that is capable of making a high‐quality bi‐directional interface with a small nerve target is urgently needed to meet the promises of bioelectronic medicine and advanced neuroprosthetics.

**Table 1:** A comparison of extraneural PNIs

Investigator	Electrode			Young's Modulus	Closure mechanism	Implant site	Nerve D [µm]	Results
	Type	Number	Material					
Tyler and Durand^[^ ^56^ ^]^	Flat interface nerve electrode (FINE)	8–12	Silicone	2 MPa	Sutures	Sciatic nerve, cat	3000	Nerve reshaping, stimulation evoked (1 d)
Foldes et al.^[^ ^57^ ^]^	Circumpolar cuff electrode	3–6	Silicone	2 MPa	Sutures	Sciatic nerve, rat	1000–1500	Conduction blocked, stimulation evoked, acute recording
Kang et al.^[^ ^58^ ^]^	Cuff electrode	16	Parylene‐C	3 GPa	Self‐closing (curl)	Sciatic nerve, rat	1000–1500	Acute recording
Plachta et al.^[^ ^59^ ^]^	Multichannel cuff electrode (MCE)	24	Polyimide	4 GPa		Vagus nerve, rat	400	Blood pressure control, stimulation evoked, acute recording
Yu et al.^[^ ^60^ ^]^	Cuff electrode	3	Parylene‐C	3 GPa	Self‐locking	Sciatic nerve, rat	1000–1500	Stimulation evoked, acute recording, chronic implantation to assess FBR (11 wk)
Park et al.^[^ ^61^ ^]^	Drug loading cuff electrode	4	Polyimide	4 GPa		Sciatic nerve, rat	1000–1500	Ex vivo: stimulation evoked, acute recording
Lancashire et al.^[^ ^62^ ^]^	Microchannel neural interface (MNI)	6	polydimethylsiloxane (PDMS) & metal foil	1–10 MPa	Sutures	Sciatic nerve, rat	1000–1500	Observed nerve regeneration, no recording/stimulation (2 mo)
Xiang et al.^[^ ^63^ ^]^	Neural ribbon (NR) electrode	8	Polyimide	4 GPa	Sutures	Peroneal, tibial, & sural nerves, rat	250–600	Stimulation evoked, acute recording
Lee YJ et al.^[^ ^64^ ^]^	Cuff electrode	4	Polyimide	4 GPa		Sciatic nerve, rat	1000–1500	Stimulation evoked, chronic charge injection via Pt black electrodes (15 wk)
Seki et al.^[^ ^65^ ^]^	Cuff electrode	10	Parylene‐C	3 GPa	Hook & loop	Sciatic nerve, mouse	1000	Hook & loop strength confirmed over 10 cycle tests, stimulation evoked
Sebetian et al.^[^ ^66^ ^]^	Cuff electrode	3	Silicone	2 MPa		Posterior tibial nerve, rat	4000–7000	External noise reduced, stimulation evoked, acute recording (1 d)
Lissandrello et al.^[^ ^67^ ^]^	Nanoclip cuff electrode	2	Carbon nanotube fibers	1 GPa	Hinged trap doors	Tracheosyringeal nerve, zebra finch	150	Stimulation evoked, subchronic recording (4 wk), latch secure (74 d)
Lee S et al.^[^ ^68^ ^]^	Sling electrode	6	Polyimide	4 GPa	Sutures	Sciatic nerve, rat	1000–1500	Stimulation evoked, stimulation powered by TENGs
Lee S et al.^[^ ^69^ ^]^	Split ring electrode	4	Polyimide	4 GPa	Split ring	Sciatic nerve, rat	1000–1500	Stimulation evoked, acute recording
Lee S et al.^[^ ^70^ ^]^	Flexible neural clip (FNC)	2	Polyimide	4 GPa	Paper clip design	Pelvic, vagus, & sciatic nerves, rat	250–400	Stimulation evoked for all nerves, wireless stimulation of pelvic nerve
Michoud et al.^[^ ^71^ ^]^	Optocuff	1	Silicone & PDMS	2 MPa	Sutures	Sciatic nerve, mouse	800–1000	Optogenetic modulation, optical stimulation evoked
Kim et al.^[^ ^72^ ^]^	Cuff electrode	4	Polyimide & PEG hydrogel	4 MPa		Sciatic nerve, rat	1000–1500	Stimulation evoked, chronic recording (5 wk)
Caravaca et al.^[^ ^26^ ^]^	Cuff electrode	16	Parylene‐C	3 GPa	Sutures	Vagus nerve, mouse	350–380	Stimulation evoked, acute recording, chronic implantation to assess FBR (12 wk)
Ong et al.^[^ ^73^ ^]^	Cuff electrode	5	Parylene‐C	3 GPa	Hydrogel adhesion	Dorsal root ganglia, cat		Acute hydrogel adhesion, acute recording
Tian et al.^[^ ^74^ ^]^	Cuff electrode	16	Parylene‐C	3 GPa	Stitches	Sciatic nerve, rat	800–1000	Stimulation evoked
González‐González et al.^[^ ^75^ ^]^	Multielectrode soft cuffs (MCS)	4–16	Thiol‐ene/acrylate polymer	550 MPa	Sutures	Pelvic & sciatic nerves, rat	200–1000	Stimulation evoked, acute recording, recording capability 30 d post‐surgery
Decataldo et al.^[^ ^76^ ^]^	Stretchable cuff electrode	2	PDMS	2 MPa	Silicone elastic seal	Renal nerve, rat	1000	Chronic recording
Elyahoodayan et al.^[^ ^77^ ^]^	Lyse‐and‐attract cuff electrode (LACE)	8	Parylene‐C	3 GPa	Locking mechanism	Sciatic nerve, rat	1000–1500	Microfluidic channels, stimulation evoked, acute recording
Otchy et al.^[^ ^20^ ^]^	Nanoclip peripheral nerve interface	6	Thin‐film polyimide	2–3 GPa	Hinged trap doors	Tracheosyringeal nerve, zebra finch	150	Spontaneous acute stimulation, chronic recording (30 d)
Song et al.^[^ ^78^ ^]^	Adaptive self‐healing electronic epineurium (A‐SEE)	3	Self‐healing polymer (SHP)	270 kPa	Self‐bonding	Sciatic nerve, rat	1000	Stimulation evoked, chronic recording (7wk), nerve‐to‐nerve interfacing
Lienemann et al.^[^ ^18^ ^]^	Stretchable cuff electrode	2	PDMS	2 MPa		Sciatic nerve, rat	1600	Conformable to nerve, stimulation evoked
Cho et al.^[^ ^29^ ^]^	Double clip neural interface (DCNI)	4	Shape memory polymer	400 MPa	Self‐clipping	Common peroneal & pelvic nerves, rat	100–200	Stimulation evoked, acute recording
Rowan et al. (This work)	Microclip peripheral nerve interface (μcPNI)	6	PDMS	2 MPa	Hinged clip	Tracheosyringeal nerve, zebra finch	150	Stimulation evoked, chronic recording (24 d)

**Figure 1:**
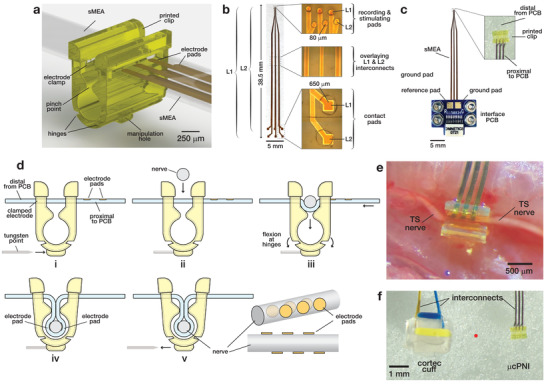
The μcPNI—a soft, flexible interface for small peripheral nerves. a) Rendering of the microclip with sMEA showing key components and features. b) sMEA component of the μcPNI electrode array after fabrication with segment descriptions. c) Photograph of the μcPNI after fabrication with sMEA compression bonded between PCBs with Omnetics connector. d) Schematic of the μcPNI implantation process: i) a sharpened tungsten point is inserted in the manipulation hole; ii) using the point , the μcPNI is advanced toward the nerve; iii) the nerve contacts the sMEA, elastically deforming the hinged uprights and allowing the electrodes to move into the microclip; iv) the sMEA wraps around the nerve as it enters the microclip; v) the point is retracted from the manipulation hole. Inset shows isometric and top views of nerve (gray) and relative electrode pad positions (yellow) after implant. e) photomicrograph of the μcPNI implanted on the TSN of a zebra finch. f) Photomicrograph of the μcPNI (right) and a Cortec silicone nerve cuff (left) each sized for 150 µm nerves. For comparison, the red circle shows the diameter of the 150 µm nerve.

Here, we present a novel PNI that meets the needs for advanced neuromodulation and integrates a 2‐photon 3D‐printed^[^ ^79^, ^80^ ^]^ microclip with a stretchable microelectrode array (sMEA). This microclip PNI (μcPNI) (**Figure** **1**a) builds on a previous design that demonstrated chronic recordings from a small nerve.^[^ ^20^, ^67^, ^81^ ^]^ However, previous designs did not enable conformal, circumferential, and high‐tissue compliance because the PI‐based electrodes^[^ ^20^ ^]^ were stiff and ultimately electrically failed due to body‐dynamics‐induced bending strain and fatigue. The next generation of the μcPNI reported here utilizes a soft and stretchable electrode array and is the first PNI with leads routed on two separate layers for high resolution and conformal recording and stimulation across the entire circumference of the nerve, providing improved electrical access and specificity. The polydimethylsiloxane (PDMS) sMEA has a Young's Modulus of 2 MPa, which reduces the mismatch in biomechanical properties with the nerve tissue compared to other materials. The microclip securely anchors the electrode array on the nerve without the need for sutures or adhesives, and allows for arbitrary placement of electrode pads around the circumference of the nerve. In addition, the printed microclip can adjust to ≈10% variations in nerve diameter through the addition of elastic hinges in the printed structure. This is important for accommodating both natural, subject‐to‐subject variation in anatomy and changes in nerve size due to post‐implant inflammation/swelling, disease processes, and subject/nerve growth. Importantly, the μcPNI is significantly smaller in size to minimize progressive neuropathy caused by stretching or rubbing of the nerve or surrounding tissue,^[^ ^82^ ^]^ while having more electrodes than commercial cuff‐type electrodes. Here, we report full electromechanical evaluation of the μcPNI and functionally validate acute and chronic recording and stimulating capabilities in vivo on the tracheosyringeal nerve (TSN) of the zebra finch, a songbird. We anticipate that the μcPNI will enable new studies of PNS function over developmental, disease, and restorative processes, and will put in reach a new generation of bioelectronic therapeutics centered on sensing and modulating peripheral circuit function.

## 2 Results

### 2.1 μcPNI Design and Fabrication

**Figure 2:**
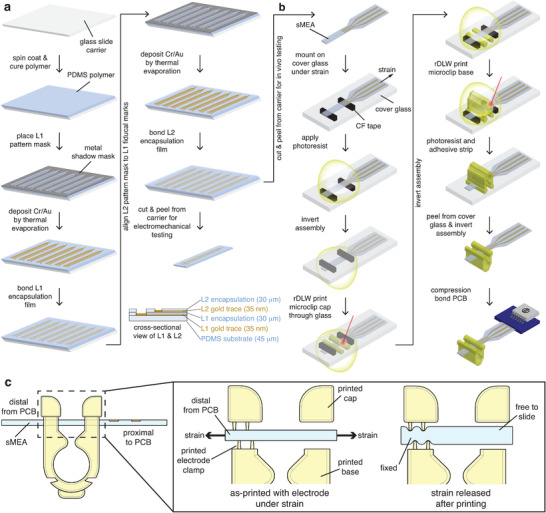
Fabrication process flow for the μcPNI. a) Fabrication of the stretchable bi‐layer PDMS electrode. b) Integration of the 3D‐printed microclip. c) Schema for microclip clamping onto the electrode: μcPNI in final assembly state (left); cross‐section of the μcPNI showing the teeth‐like printed clamp on only one side and the electrode in a pre‐strained state that reduces the thickness of the substrate (center); following release from strain, the electrode thickness is restored and now mechanically fixed in the printed clamp (right).

**Figure 3:**
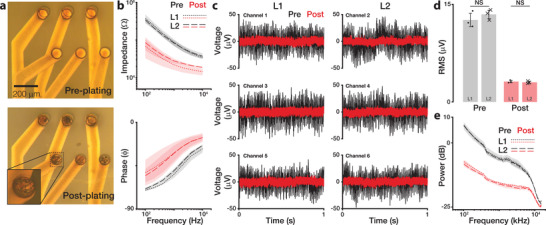
Electrochemical characterization of the electrode array. a) Photomicrographs of μcPNI electrode sites before (top) and after (bottom) electroplating with Pt black. Inset shows the zoomed view of plated electrode pad. b) Electro‐impedance spectroscopy of the six channels on the μcPNI before (black) and after (red) electroplating with Pt black. Mean of L1 and L2 electrodes shown as dotted and dashed lines, respectively; standard deviation across a layer indicated by shaded regions. c) Baseline noise recordings of the six μcPNI channels before (black) and after (red) electroplating with Pt black. d) RMS of baseline noise across all channels (n = 3 each on L1 and L2) before (black) and after (red) electroplating. Markers identify individual channels; bars and error bars denote mean ± std across n = 3 channels. N.S. = not significant (*P* = 0.4 and 0.7, respectively). e) Mean power spectrum of baseline noise across channels before (black) and after (red) electroplating. Mean of L1 and L2 pads shown as dotted and dashed lines, respectively; standard deviation across a layer indicated by shaded regions.

The μcPNI consists of three components: 1) a sMEA (Figure 1a–c), 2) a microclip (Figure 1a,c), and 3) a printed circuit board (PCB) (Figure 1c). The sMEA has six electrodes, three on Layer 1 (L1) and three on Layer 2 (L2)^[^ ^83^ ^]^ to maximize electrode density and reduce the overall dimensions of the device without compromising yield. To secure the electrode on the nerve, we developed the microclip, a 3D printed mechanical nerve anchor using resonant Direct‐Laser‐Write (rDLW) technology that rapidly structures a biostable photoresist (IP‐Dip) with ≈1 µm minimum feature sizes.^[^ ^79^ ^]^ The printed microclip consists of a U‐shaped channel comprising a retention cavity that retains the electrode during implant and hinges that allow flexion under µN‐scale forces (Figure 1a,d). Under force applied via a tungsten surgical point inserted in the manipulation hole in the base, the microclip hinges open, allowing the soft electrode to wrap snuggly around the nerve as it moves through the pinch point and into the retention cavity. In addition to reducing the complexity of surgical manipulation, the wrap‐on‐implant concept can place the electrode pads at arbitrary points on the circumference of the nerve (Figure 1d, lower right) and eliminates the need for suturing or surgical adhesives to stabilize the preparation. This design and implantation method enabled significant miniaturization, keeping the overall scale of the μcPNI (800×500×800 µm) comparable to that of the implant target (here, the 150 µm diameter songbird TSN, Figure 1e) and more than an order of magnitude smaller than commercially available PNIs (Figure 1f).

To create the mechanically stable yet elastic electrode array of the μcPNI, we developed the fabrication procedure schematized in **Figure** **2**a (see Experimental Section). The bi‐layer sMEA consists of 5 layers: 1) the PDMS substrate (45 µm), 2) the microcracked gold^[^ ^84^, ^85^ ^]^ electrodes of L1, 3) the PDMS encapsulation of the electrodes on L1 (30 µm), 4) the microcracked gold^[^ ^83^ ^]^ electrodes of L2, and 5) the PDMS encapsulation of the electrodes on L2 (30 µm) (Figure S1, Supporting Information). The recording sites of the electrodes (80 µm diameter) are connected via electrode leads (width: 100–380 µm) to the contact pads (width: 650 µm) of the PCB. The finished electrodes were laser‐cut and removed from the glass carrier with blunt forceps (Figure S2, Supporting Information). To facilitate the electrical connection to external test equipment, the contact pads on the sMEA were compression bonded to a connectorized PCB; silver paste was applied to the contact pads to ensure a low‐impedance junction.

The μcPNI required a novel process for robust integration of the soft, elastic electrode with the printed microclip (Figure 2b; see Experimental Section for additional printing details). Prior studies used residual surface tension,^[^ ^86^, ^87^, ^88^ ^]^ post‐printing deposition of adhesives and coatings,^[^ ^67^, ^89^ ^]^ or lock‐and‐key‐like construction methods^[^ ^20^, ^81^ ^]^ to affix printed microstructures to functional substrates. However, these established methods were incompatible with the need to maintain electrode flexibility and motility within the microclip during implantation (Figure 1d).

Instead, we leveraged the Poisson compression of PDMS under strain to create a printed mechanical clamp of the electrode (Figure 2c). Briefly, the stretchable electrode was mounted under longitudinal tension onto a cover glass carrier with carbon fiber tape (Figure 2b). The applied strain reduced the thickness of the electrode consistent with the Poisson effect, and the microclip cap and base were printed on opposing sides of the tensioned electrode. Critically, only one side of the cap contains an electrode clamp printed flush with the surface of the tensioned electrode (Figure 2c, center). Following printing, the entire assembly was submerged in nitromethane to strip both the unpolymerized photoresist and the mounting adhesive, releasing the electrode from tension. Upon strain relaxation and reversal of Poisson compression, the electrode was thicker than the gap between opposing sides of the printed clamp (≈60 µm), and the electrode was mechanically fixed at the end distal to the PCB while the proximal segment remained free to slide within the microclip (Figure 2c, right). Thus, the rigid microclip can be robustly attached to the soft electrode without the need for additional manual assembly or post‐printing adhesive application.

### 2.2 Electromechanical Characterization

Electrical and mechanical stability of the μcPNI is critical for in vivo recording and modulation of small nerves within a biomechanically dynamic environment. During implantation in vivo, the leads of the μcPNI experience static bending strain in the section that is in contact with the microclip, and multi‐axial mechanical deformation in the section between the microclip and the PCB in response to the songbird's natural body movements. It is important that these mechanical deformations do not affect the impedance, hence recording and stimulation properties, of the electrodes. We performed benchtop testing of μcPNIs to assess their potential for safe and effective chronic implantation in small animals, with a particular emphasis to detect differences between electrodes on the two layers.

#### 2.2.1 Electrical Characterization

As‐fabricated electrodes have a high impedance (>1 MΩ) due to their small surface area (≈5000 µm^2^), which contributes to higher noise for recordings and artifacts for stimulation. To reduce electrode impedance, platinum black was electrodeposited on the electrode surface (**Figure** **3**a; see Experimental Section). As described previously,^[^ ^90^ ^]^ the platinum black does not cover the entire recording sites of the microcracked electrodes evenly, but produces a distinct pattern. Platinum black grows preferentially along larger cracks in the gold film which are caused by coalescing microcracks. The microcracks coalesce due to small tensile strains during peeling of the μcPNI from the glass backing plate after fabrication and by manually handling. The preferential growth of platinum black along large cracks may be caused by a lower overpotential for the reduction of the [PtCl_6_]^2−^ ions because the gold at recently coalesced cracks has not been directly exposed to post‐metal deposition processing.

Impedance spectroscopy of the electrodes before and after plating showed a larger than tenfold reduction in impedance (at 1 kHz) across all electrodes on both L1 and L2 layers from 465 ± 200 to 20 ± 7 kΩ (Figure 3b) with no significant difference between electrode layers pre‐ and post‐plating (*P* = 0.3 and *P* = 0.7, respectively). The comparable measurements of the two electrode layers (*P* = 0.1) suggest that a slight misalignment (≈10 µm, Figure 3a top) between electrodes and encapsulation layer openings will have no appreciable effect on impedance and recording measurements. Indeed, it is critical that a small misalignment does not cause appreciable differences in electrode impedance because it is technically very challenging to align and bond PDMS‐based multi‐layer devices with high accuracy over large distances (here: >40mm from recording sites to contact pads to PCB). The reduction in impedance was accompanied by a reduction in peak‐to‐peak voltage (*V*_PP_) noise from >60 µV to <10 µV (bandwidth: 0.001–7.5 kHz; Figure 3c) that was consistent across channels and not significantly different between electrode layers (*P* = 0.7) (Figure 3d). This baseline noise is desirable as the nerve signals are in the range of tens to hundreds of µV. Comparison of the mean power spectrum of baseline noise before and after electroplating revealed a broad decrease in noise power in the 0.5–6 kHz frequency band that is associated with multi‐unit neuronal activity (Figure 3e), again without any significant difference between electrodes on both layers.

#### 2.2.2 Electromechanical Stability under Bending Strains

The bending strain (*ε*) in the electrodes is proportional to the distance of the gold film from the neutral plane, *d*_N_, and inversely proportional to the bending radius, BR (*ε* = *d*_N_ BR^−1^). In the microclip section of the μcPNI, the bending strain is highest due to the small BR when the leads are wrapped around small nerves. To reduce the bending strain, we chose the thickness of the PDMS substrate and the encapsulation layers for L1 and L2 so as to minimize *d*_N_. Furthermore, the bending strain on the gold film is in the microclip section is compressive by design, and previous research has demonstrated that the resistance of single layer microcracked gold conductors is not appreciably altered during static bending for strains up to at least 15% for electrode leads in compression.^[^ ^91^ ^]^ We have also measured the impedance of all six electrodes on several μcPNIs pre‐plating, post‐plating, in vivo while implanted, and post‐explantation (Figure S3, Supporting Information). The electrode impedance is not appreciably altered after three weeks of implantation, that is, the impedance post‐plating (20±7 kΩ) and post‐explantation (29±10 kΩ) are comparable. The impedance in vivo is higher (75±13 kΩ) compared to before implantation (post‐plating) and post‐explantation, but remains below 100 kΩ for all electrodes. The difference in ionic strength of the medium and the confinement of the recording sites between the microclip and the nerve likely contribute to this increase in impedance.

**Figure 4:**
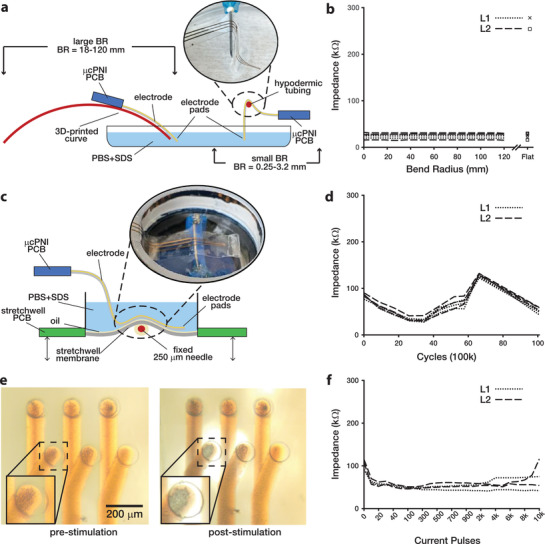
Electromechanical characterization of the electrode array. a) Experimental setup to assess bending strain. b) Electrode impedance versus bending radius; L1 and L2 pads shown as dotted and dashed lines, respectively; markers identify individual channels. c) Experimental setup to assess bending fatigue. d) Electrode impedance versus bending cycles; L1 and L2 pads shown as dotted and dashed lines, respectively. e) Micrographs of μcPNI electrode recording/stimulation sites before (left) and after (right) injecting 10 000 pulses at 110 µA and 133 µs. f) Impedance versus number of stimulation pulses. Note the non‐uniform x‐axis.

In the section of the μcPNI between the microclip and the PCB, the electrode leads can be in tension or compression depending on the bending direction induced by body dynamics. In tension, the resistance of microcracked conductors increases exponentially with bending strain, and bending causes a larger increase in resistance for the same strain compared to stretching.^[^ ^91^ ^]^ For this reason, the gold electrodes on the μcPNI are, by design, close to the neutral plane (to minimize strain) and in compression (L2 only) when wrapped around the nerve. To validate the robustness of the bi‐layer μcPNI, the array was bent in tension at BR ranging from 250 µm to 120 mm while the impedance at 1 kHz was measured in phosphate‐buffered saline (PBS) and sodium dodecyl sulfate (SDS; 40 mM, Sigma Aldrich) (**Figure** **4**a). The test was performed four separate times and averaged for each channel. Figure 4b shows a plot of the impedance of all six channels versus the BR. The impedance for a given channel does not change appreciably with BR. Importantly, there is no significant difference between electrodes on L1 and L2 (one‐way ANOVA: *P* = 0.51; see Experimental Section for details on this and all other statistical analyses).

#### 2.2.3 Electromechanical Stability under Bending Fatigue

Previous research has shown that single layer microcracked gold electrodes can be stretched by more than 20% for over a million cycles.^[^ ^92^, ^93^ ^]^ In this work, we assessed the effects of bending fatigue on the impedance of a bi‐layer μcPNI. The leads of the electrodes were continuously wrapped and unwrapped over 1 Million cycles around a 250 µm radius hypodermic needle, and the electrode impedance (at 1 kHz) was measured periodically (Figure 4c). A BR of 250 µm was chosen because the electrodes are unlikely to experience higher bending strain (smaller BR) in the section between the microclip and the PCB during implantation. Figure 4d shows a plot of the impedance of all six channels versus the number of bending cycles. There is no appreciable bending fatigue over the course of 1 Million bending cycles as no sustained increase in impedance is observed. The variation in impedance over 1 Million bending cycles (90 kΩ ± 35 kΩ) are random measurement variances that are difficult to avoid with the sample being located in an incubator for two weeks. Importantly, we found no significant difference in the impedance of electrodes on different layers at any time point (one‐way ANOVA: *P* = 0.078; see Experimental Procedures).

#### 2.2.4 Charge Injection Limits

The charge injection limit is the maximum amount of charge (current × time) that can be injected without causing irreversible damage to the electrode. To assess the charge injection limit for the μcPNI, a 110 *μ*A constant current bi‐phasic stimulus (at 1 Hz and 133 µs phase^−1^) was generated out‐of‐phase by two of six Pt Black‐coated μcPNI electrodes (80 µm diameter; 22 mA mm^−1^ charge density) in a PBS solution. 10000 biphasic, bipolar stimulating pulses were first injected through two L1 electrodes and then two L2 electrodes for a total of 20 000 pulses. Figure 4e shows the recording/stimulation sites before and after 10 000 stimulation pulses with minor metal degradation in the observed electrode. Figure 4f shows the impedance (at 1 kHz) of the four stimulated electrodes in PBS over the course of 10 000 stimulation pulses. The impedance of all electrodes decreases from about 100 kΩ to about 50 kΩ over the first 20 stimulation pulses presumably due to removal of contamination from the electrode surface by desorption or oxidation of contaminants caused by the current pulses. After the first 20 pulses, the impedance of electrodes on L1 and L2 remained constant over 10 000 pulses despite the observed degradation, and we again found no significant difference in the impedance of electrodes on different layers at any time point (one‐way ANOVA: *P* = 0.074; see Experimental Section).

Initial testing injected 200 μA constant current bi‐phasic stimulus (at 1 Hz and 200 μs phase^−1^; 40 mA mm^−1^ charge density) through μcPNI L1 and L2 electrodes to better investigate the in vivo stimulation conditions of this study. Electrodes in these tests demonstrated significant metal degradation and increases in impedance between 5000–10 000 pulses. It should be noted that, in future clinical applications, the size of the stimulating electrodes will be significantly larger (i.e., the current density will be lower), thus increasing the number of pulses that can be injected without damaging the electrodes.

### 2.3 In Vivo Functional Validation

To validate the performance of the μcPNI in sensing and modulating small nerve activity, we implanted the μcPNI on the zebra finch TSN—an avian hypoglossal analog that innervates the songbird vocal organ (i.e., the syrinx).^[^ ^94^ ^]^ The TSN is an ideal model in which to characterize PNI technologies due to its surgical accessibility and physiological homologies to mammalian sensorimotor nerves of therapeutic interest.^[^ ^67^, ^95^ ^]^ In addition, the multi‐month stereotypy of singing‐related TSN activity patterns and their high temporal correlation with vocalization provide a strong benchmark against which to assess chronic performance and stability.^[^ ^20^, ^81^ ^]^

#### 2.3.1 Acute Recording

**Figure 5:**
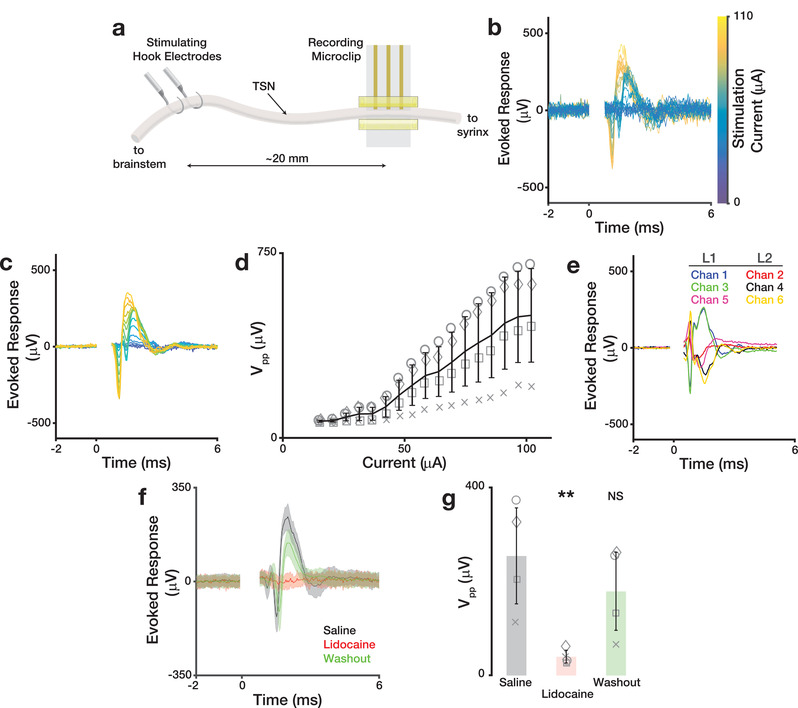
Acute in vivo recording of stimulation‐evoked nerve activity. a) Schema for acute recording of evoked responses. Current‐controlled stimulation was delivered via bipolar silver hook electrodes; evoked responses were recorded by a μcPNI placed ≈20 mm caudally. b) Representative examples of responses evoked by graded stimulation currents. Stimulation (10–110 µA; biphasic pulses; 200 µs phase^−1^) applied at t = 0 ms. Each line shows the single‐trial response; line color indicates stimulation current as indicated in the color bar. Note that for clarity of presentation and to facilitate comparison of the pre‐ and post‐stimulation waveforms, the stimulation artifacts have been removed from this and other subpanels in this figure. c) Same as in (b), but showing mean response across trials (16–64 trials) at binned stimulation currents (bin width = 5 µA). Each line shows the mean response; line color indicates stimulation current as in (b). d) Evoked response peak‐to‐peak voltage (*V*_pp_) as a function of stimulation intensity. Each data point indicates the mean across trials within an animal (16–64 trials each). Gray symbols identify individual birds; black lines and error bars indicate the mean and standard deviation across animals (n = 4 birds). e) Representative examples of evoked response waveforms recorded simultaneously on each electrode. Stimulation (67 µA; biphasic pulses; 200 µs phase^−1^) applied at t = 0 ms. Each line shows the mean response across trials (n = 64 trials); line color indicates the recording channel. f) Example of evoked responses recorded before, during, and after local lidocaine application. Stimulation (64 µA; biphasic pulses; 200 µs phase^−1^) applied at t = 0 ms. Each line and shaded region show mean ± std for n = 24 trials. g) Evoked response *V*_pp_ across conditions in (f). Each data point indicates the mean across 24 trials within an animal. Grey symbols identify individual birds; bars and error bars denote mean ± std across n = 4 birds per condition. **P < 0.01 Repeated‐measures ANOVA, *P* = 0.007; Dunnett's test, *P* = 0.009.

To assay μcPNI acute recording performance, we recorded evoked compound responses from the TSN in anesthetized zebra finches. Two ≈2 mm segments of the right‐side TSN approximately 20 mm apart were dissected from surrounding tissue. A μcPNI was implanted on the caudal segment and bipolar silver hook electrodes were placed at the rostral location (**Figure** **5**a). Biphasic stimulation pulses (200 µs phase^−1^) were delivered at 1 Hz, and evoked response voltages were recorded simultaneously for all six channels. Consistent with prior studies^[^ ^20^, ^67^, ^81^ ^]^ and estimates of conduction velocities in 5–8 µm nerve fibers,^[^ ^96^ ^]^ we identified the primary components of evoked responses 0.75–4 ms after stimulation onset. We obtained graded evoked response curves by varying the stimulation current amplitude (Figure 5b,c). Across experiments in n = 4 birds, *V*_pp_ showed a sigmoidal relationship with increasing stimulation current (Figure 5d), consistent with standard models of fiber recruitment.^[^ ^97^, ^98^ ^]^ Furthermore, we observed that the common mode subtracted response waveforms were unique across channels (Figure 5e). This uniqueness stands in marked contrast to prior studies showing highly correlated waveforms across closely spaced channels on one side of an implanted nerve^[^ ^20^, ^26^, ^75^, ^99^ ^]^ and suggests that even within this small geometry spatially distributed recording electrodes can access distinct subcomponents of the evoked response. To confirm the neuronal origin of these responses, we applied 2% lidocaine at the stimulating site to reversibly block nerve conduction (Figure 5f). Across experiments (n = 4 birds; Figure 5g), lidocaine abolished evoked responses with *V*_pp_ declining significantly from saline controls (*P* = 0.009); subsequent washout with saline restored response amplitudes to not significantly different from control (*P* = 0.29).

#### 2.3.2 Chronic Recording

**Figure 6:**
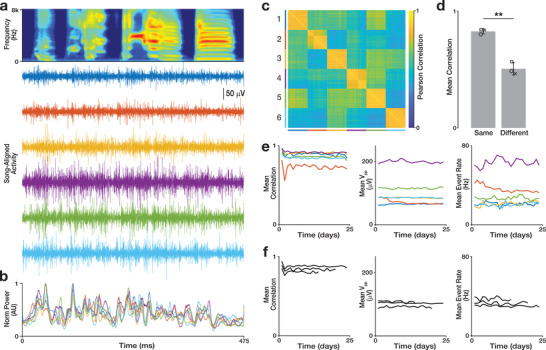
Chronic in vivo recording of nerve activity. a) Representative example of chronic TSN recording aligned to the song. (Top) Spectrogram of the bird's song; color indicates power intensity at each time‐frequency bin. (Bottom) Electrophysiology activity recorded from the TSN simultaneously with the song motif shown at top; timescale aligned to Figure 6b at the bottom. Color here and in all other subpanels indicates the recording channel. b) Mean song‐aligned TSN activity envelope over n = 10 consecutive motifs for each channel. Same animal as shown in (a). c) Matrix of pair‐wise correlations between mean song‐aligned activity envelopes from each channel (n = 50 each channel; 300 total) for the representative animal shown in (a). Row and column relations to channel indicated by colored lines at left and bottom. d) Mean pair‐wise correlation between nerve activity envelopes recorded within (left) and across (right) μcPNI electrode in n = 3 birds. In each, data points show mean across electrodes within a bird. Bars show mean across all birds; error bars indicate std. **P < 0.01 two‐tailed paired t‐test, *P* = 0.008. e) Metrics demonstrating stable performance of each μcPNI channel over time. (Left) Mean daily trial‐by‐trial Pearson's correlation to the average activity pattern on the 1st day of recording. (Center) Mean daily peak‐to‐peak voltage. (Right) Mean daily event rate. f) Same as in (e), but showing mean statistics across all electrode channels in n = 3 birds (two‐tailed paired t‐test; correlation: *P* > 0.17; *V*_pp_: *P* > 0.68; event rate: *P* > 0.37).

Though acute studies are critical for probing PNS physiology, such experiments provide only a brief snapshot of nerve function and fail to address how the function is modulated in other contexts^[^ ^100^, ^101^, ^102^, ^103^, ^104^, ^105^ ^]^ or by slower developmental, disease, or restorative processes.^[^ ^1^, ^11^, ^90^, ^106^, ^107^, ^108^ ^]^ To validate the device for chronic recordings from a small nerve, we implanted the μcPNI on the songbirds TSN, the primary output of the singing‐related central neural circuits and the sole source of innervation to the syrinx, and recorded singing‐related nerve activity from tethered freely moving birds (n = 3; **Figure** **6**).

All animals showed normal behavior with usual food intake, unencumbered movement, and resumption of spontaneous singing within 2 days of the implant. There were no signs of pain, distress, or other impairment due to the presence of the implant. From the first utterances, we observed robust singing‐related multi‐unit activity with amplitude modulations of up to 200 µV on all six electrode channels. These signals survived common‐mode subtraction and filtering (Figure 6a), supporting their neuronal (vs artifactual or electromyographic) origin.^[^ ^20^, ^109^ ^]^ Consistent with prior studies^[^ ^20^, ^110^, ^111^, ^112^ ^]^ of song‐related neuronal activity, we found that stereotyped segments of song (Figure 6a, top) were reliably associated with signal envelopes exhibiting similar time‐varying trajectories. Furthermore, we found that daily mean signal envelops were broadly correlated across channels; however, these signal envelopes also showed small but reliable deviations at discrete timepoints (Figure 6b). To quantify these similarities, we calculated the Pearson correlation between song‐aligned activity envelopes from each channel (n = 500 trials across six channels in one bird; 3000 signal envelopes in total) (Figure 6c). Across experiments, we found that signal envelopes recorded on the same electrode were significantly more correlated than those recorded on adjacent electrodes (n = 3 birds, *P* = 0.008; Figure 6d). This finding echoes a similar observation in the acute experiments (Figure 5e) and further suggests that the spatially distributed electrodes may be sampling unique subcomponents of TSN activity.

We recorded singing‐related TSN activity in unrestrained zebra finches for up to 24 days (n = 3 birds, range: 14–24 days). Over the duration of the experiments, the recordings showed well‐defined signals on each channel and a remarkable degree of stereotypy in song‐aligned activity envelopes. To quantify the stability of these recordings over time, we calculated trial‐by‐trial for each channel the Pearson correlation between song‐aligned TSN activity envelopes and the mean envelope on day 1 (Figure 6e, left), the *V*_pp_ of singing‐related activity (Figure 6e, center), and the mean spike event rate (Figure 6e, right). Across n = 3 birds, we found no significant differences in the daily means of these metrics across channels between day 1 and the last day of recording (correlation: *P* > 0.17; *V*_pp_: *P* > 0.68; event rate: *P* > 0.37; Figure 6f). These analyses demonstrating stable longitudinal recordings of TSN activity suggest that the μcPNI is well‐suited to recording PNS function at chronic timescales.

#### 2.3.3 Acute Stimulation

**Figure 7:**
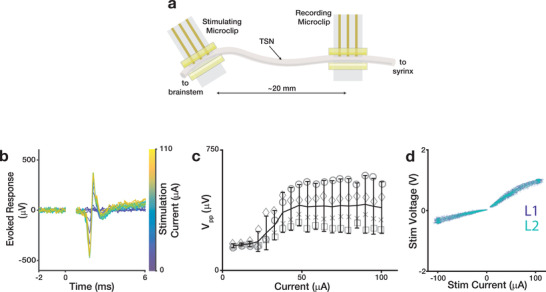
Acute in vivo stimulation of nerve activity. a) Schema for acute stimulation of evoked responses. Current‐controlled stimulation was delivered via μcPNI; evoked responses were recorded by an additional μcPNI placed ≈20 mm caudally. b) Representative examples of responses evoked by graded stimulation currents. Stimulation (10–110 µA; biphasic pulses; 200 µs phase^−1^) applied at t = 0 ms. Each line shows mean response across trials (8–40 trials) at binned stimulation currents (bin width = 5 µA). Line color indicates stimulation current as indicated in the color bar. c) Evoked response peak‐to‐peak voltage (*V*_pp_) as a function of stimulation intensity. Each data point indicates the mean across trials within an animal (8–40 trials each). Gray symbols identify individual birds; black lines and error bars indicate the mean and standard deviation across animals (n = 4 birds). d) Summary figure of stimulation voltage as a function of stimulation current across experiments (11 774 trials across experiments using n = 4 μcPNI). Pulses delivered by L1 and L2 electrode pads indicated by color.

To assay acute stimulating performance in vivo, we used a two‐interface preparation that allowed us to record responses with one μcPNI that were evoked via the second μcPNI placed ≈20 mm caudally (**Figure** **7**a). Biphasic, current‐controlled stimulation pulses (200 µs phase^−1^) were delivered at 1 Hz via two electrode sites (e.g., 1 most‐caudal and 1 most‐rostral electrode) at the caudally implanted μcPNI, and response voltages were recorded at the rostral μcPNI (see Experimental Section). We obtained graded evoked responses by varying the stimulation current (Figure 7b). As expected, the *V*_pp_ of the responses showed the canonical sigmoidal relationship with stimulation current (n = 4 μcPNIs; Figure 7c). Intriguingly, we found that the recruitment curve plateaued at ≈50 µA—less than half the stimulating intensity at which the bipolar hook electrode evoked responses similarly saturated (Figure 5d). This lower saturation limit is evidence that the enhanced electrical isolation of the μcPNI stimulation sites provides more efficient depolarization of the tissue compared to the exposed hook electrodes. An alternative explanation is that the μcPNI electrodes were unable to deliver the commanded charge injection.^[^ ^113^ ^]^ To rule out this alternative explanation, we verified the magnitude of stimulation by monitoring the command current and voltage delivered by the stimulator to the electrode pad at each phase of the pulse (Figure 7d). This analysis revealed that over the range of command currents (−100 to 100 µA; n = 11 774 pulses), the stimulating voltage remained well below the stimulator maximum (10 V) and showed no discontinuities, indicating an electrochemical limit had not been reached. In addition, the maximum evoked response in the stimulus‐response curve for μcPNI (Figure 7c) and hook stimulating electrodes (Figure 5d) is of comparable magnitude, indicating that all fibers are stimulated with half the current in the μcPNI compared to hook electrodes. Furthermore, we found no significant difference in the current‐voltage relationship for stimulating pulses mediated by electrodes on L1 of the μcPNI versus those on L2 (*P* = 0.53). Taken in total, these results suggest that the μcPNI is well suited for full‐duplex acute interfacing with small peripheral nerves.

## 3 Discussion and Conclusion

Implantable neural interfaces are engineered systems designed to study and treat the nervous system. Cochlear implants restore hearing in deaf children, deep brain stimulation alleviates Parkinsonian symptoms, and spinal cord neuromodulation attenuates neuropathic pain. In addition, it is thought that a broad range of diseases may be treatable through precise intervention in the PNS.^[^ ^4^, ^11^, ^114^ ^]^ The hope for such bioelectronic therapeutics has prompted the development of devices targeting a variety of nerves and ganglia throughout the PNS. However, persistent challenges bridging the mismatch in mechanical properties and scale have hindered the realization of PNIs mediating long‐lasting interfacing with small nerves.

To address these challenges, we developed the μcPNI (Figure 1)—a microscale nerve interface that combines a soft, stretchable electrode array with a 3D‐printed nerve anchor (Figure 2). We demonstrate that the μcPNI provides a stable bioelectronic interface under deformation and mechanical strain comparable to those inflicted on implants by body movement (Figures 3 and 4). In addition, we showed that the μcPNI can make stable, high‐quality recordings of a small nerve in a tethered freely moving animal over multi‐week timescales (Figures 5 and 6). Finally, we demonstrated that the μcPNI can achieve graded neuromodulation of a small nerve (Figure 7). To our knowledge, a chronic format PNI with comparable size, performance, and similar biomechanical compliance has not been described previously. This device provides a new reference for the safe implant, stable high‐quality recording over multi‐week timelines, and graded modulation of small peripheral nerves.

The ability to capture robust signals in chronic experiments highlights the quality of our stretchable, low impedance interface. In comparison to traditional PNI formats (Table 1), we see two main advantages of our approach: first, the compact design and the possibility to easily adapt the shape facilitate the surgical procedure alleviating the need to position and suture individual microwires. Second, we suggest that the stretchable properties of the electrode and its interconnect are important to achieve long‐term recordings. Like many peripheral nerves, the TSN runs along the trachea distant from any rigid support tissue and during animal movement the area is subjected to significant motion and displacements. In traditional PNIs made from rigid bulky materials, body dynamics are related to device failure in chronic conditions occurring a few days after surgery. Instead, our stretchable electrode and microclip can adapt to the dynamic environment without generating interfacial forces that potentially damage or displace the device.

Though our efforts here have been focused on creating an implantable device for bioelectronic interfacing for small nerves, the materials, microclip design concept, and microfabrication techniques developed for the μcPNI could serve as a useful technology platform for other soft medical electronics. Securely placing implantable sensors on or nearby the soft, delicate tissues of the body is a major scientific and engineering challenge—for example, in the study of the vasculature, secretory organs, and ducts, or other active tissues—thus we believe this technology has the potential to impact biomedical research and tool development broadly.

## 4 Experimental Section

### Fabrication of Microelectrode Arrays

The fabrication of bi‐layer sMEAs of the μcPNI builds and extends on previously described methods^[^ ^83^, ^93^, ^95^, ^115^, ^116^ ^]^ (Figure 2a; see Supporting Information for details). Briefly, the microcracked gold film for the L1 electrodes was deposited by sequential thermal evaporation of 3 nm Cr, 35 nm Au, and 3 nm Cr thickness through a shadow mask on a 45 µm thick PDMS membrane on a glass carrier. The L1 structures were encapsulated by transfer bonding with a 30 µm thick PDMS layer with pre‐patterned holes exposing the electrode and contact pads. The L2 structures were similarly defined in gold and chromium via shadow masks and encapsulated by a second pre‐patterned 30 µm thick PDMS layer. Figure 1b shows the details of the μcPNI array after fabrication.

### Fabrication of the Microclip

Key steps in the fabrication of the device are overviewed in Figure 2b. The key fabrication steps were as follows: i) the stretchable electrode was mounted under tension on a thin optical glass substrate (24 mm x 60 mm, #0‐thickness cover glass, Gold Seal) with 250 µm‐thick double‐sided acrylic tape (3M), and a drop (≈0.5–2 µL) of liquid acrylic photoresist (IP‐Dip, Nanoscribe, GmbH) was deposited over and beneath the recording array; ii) the glass and sample were inverted and the microclip cap (Figure 2c) printed through the optical glass substrate using a two‐photon‐polymerization‐based, dip‐in resonant direct laser writing (rDWL) process;^[^ ^79^ ^]^ iii) the glass substrate and sample were righted, and the base of the microclip was printed with the rDWL process; iv) the photoresist was developed and tape adhesive dissolved by submerging the glass substrate, electrode, and nanoclip in nitromethane (Sigma Aldrich) for 20 min, and the whole device rinsed in methoxy‐nonafluorobutane (Novec 7100; 3M) to remove trace solvent residue; v) the assembly was removed from the glass slide and compression bonded to a connectorized PCB as described above. All mechanical design was performed with Solidworks (Dassault Systèmes). All tested and imaged devices were fabricated using a custom rDLW system.^[^ ^79^ ^]^

### Printed Circuit Board Assembly

The section of the sMEA with the contact pads (proximal section) was compression‐bonded between two custom PCBs^[^ ^83^, ^117^ ^]^ with a 16‐channel Omnetics connector (A79040‐001) (Figure 1c; see Supporting Information for details).

### Electroplating with Platinum Black

The recording sites of the electrodes were electroplated with platinum black as previously described^[^ ^83^, ^117^ ^]^ to reduce the electrode impedance (Figure S4, Supporting Information for details), and thus the noise of the recording and the size of the stimulation artifact.

### Impedance and Recording Noise Measurements

Post‐electroplating impedance and recording noise in PBS was measured (Intan RHS 128ch recording/stimulation controller, Intan Technologies) at 1 kHz against a Pt counter electrode inside a custom‐built Faraday cage with the cage connected to PCB ground (Figure S5, Supporting Information). The target impedance was 100 kΩ > Z > 1 kΩ, and electrodes were not considered functional if impedance was outside this range.

### Evaluation of Bending Strain

Impedance of all six electrodes was measured while bending the leads of the μcPNI array around spherical segments that were 3D printed with acrylonitrile butadiene styrene (ABS) for BR 18–120 mm, around the shanks of drill bits for BR 3.2–0.8 mm, and around the shaft of a hypodermic needle for BR 0.25 mm. The electrode array was plasma treated (30 s) and immersed in PBS and bent over the segment (Figure 4a). The impedance was measured (Intan RHS 128ch recording/stimulation controller, Intan Technologies) inside a Faraday cage as described above, with the cage connected to PCB ground, and a Pt counter electrode connected to PCB reference (Figure 1c). Three impedance measurements were taken and averaged for each of the fourteen bending radii ranging from 250 µm to 120 mm.

### Evaluation of Bending Fatigue

The distal end of the μcPNI (section with the recording sites) was bonded to a PDMS stretchwell with a small amount of uncured PDMS (same as for the substrate) followed by curing for at least 12 h at 60 °C. A stretchwell consists of a PDMS membrane that was sandwiched between two PCBs with circular cutouts in the center and a polycarbonate well that was glued to the top PCB (Figure S6, Supporting Information). The well was filled with PBS solution. The distal end of the μcPNI with the PCB was placed outside of the stretchwell. With this arrangement, the μcPNI was exposed to a small amount of tensile strain in addition to bending strain, which realistically reproduces the mechanical forces that the μcPNI will experience in vivo. For fatigue testing of the μcPNI array, the stretchwell was mechanically raised and lowered around the shaft of a hypodermic needle with a 250 µm radius (Figure 4c). One bending cycle comprised of wrapping and unwrapping the μcPNI leads at a 45° angle around the needle shaft. A total of 1 000 000 cycles were performed at 1 Hz and the electrode impedance in PBS was measured (Intan RHS 128ch recording/stimulation controller, Intan Technologies) periodically over the course of two weeks.

### Evaluation of Safe Charge Injection Limit

The Pt Black coated electrodes of the μcPNI were immersed in PBS and connected to an Intan RHS 128 channel recording/stimulation controller (Intan Technologies). 10 000 biphasic, out‐of‐phase bipolar stimulating pulses were injected at 1 Hz at stimulus amplitude (110 µA) and duration (133 µs). Electrode impedance was measured (Intan RHS 128ch recording/stimulation controller, Intan Technologies) before and after every 10 stimulation pulses for the first 100 pulses, after every 100 stimulation pulses up to 1000 pulses, etc.

### Vertebrate Animal Subjects

The care and experimental manipulation of the animals were performed in accordance with the guidelines of the National Institutes of Health and were reviewed and approved by the Boston University Institutional Animal Care and Use Committee (protocol numbers PROTO201800577 and PROTO201800578). Adult male zebra finches (Taeniopygia guttata; 90+ days after hatch, n = 11 birds) were obtained from the Boston University breeding facility and housed on a 13:11 h light/dark cycle in individual sound‐attenuating chambers with food and water provided ad libitum. Because the behavioral effects of the interventions could not be pre‐specified prior to the experiments, sample sizes were chosen that would allow for the identification of outliers and for experimental reproducibility. No animals were excluded from experiments post‐hoc. The investigators were not blinded to the allocation of animals during experiments and outcome assessment.

### Surgical Procedures

All surgical procedures were performed under isoflurane anesthesia (1–4% dissolved in oxygen), with peri‐operative analgesia (1% Lidocaine, SC) and anti‐inflammatory (1% Meloxicam, IM) regimens. All nerve implants reported in this study targeted the avian hypoglossal cranial nerve, the TSN. The TSN, which runs along the length of the songbird trachea and terminates at the syrinx, has a diameter of approximately 150 µm and was composed of both afferent and efferent fibers.^[^ ^67^ ^]^ At all experiment end‐points, animals were given an overdose injection of sodium pentobarbital (250 mg kg^−1^ Euthasol, IC).

### Acute Preparation (n = 8 Birds)

An anesthesia mask was placed over the bird's head and the animal was placed in a supine position with a small pillow beneath the neck for support. Feathers were removed from the lower head, neck, and upper chest, and betadine antiseptic solution (5% povidone‐iodine) and ethanol (70%) were successively applied to prepare the incision site. A 20–25 mm incision was made at the base of the neck, and the tissue blunt was dissected to expose the trachea. Sutures were placed in the skin of the lateral edge of the incision and retracted from the body to expose the implant site. Connective tissue surrounding the nerve was bluntly dissected away, and two sections of the TSN (each 3–4 mm and approximately 15 mm apart) were isolated from the trachea. A μcPNI was implanted at the rostral location. For the recording experiments reported in Figure 5 (n = 4 birds), a bipolar silver hook stimulating electrode was placed at the caudal location; for the stimulating experiments reported in Figure 7 (n = 4 birds), a second μcPNI interface was implanted at the caudal location. For all recording experiments, a platinum reference wire (0.003 in dia., Teflon coating; AM‐Systems) with tip exposed was sutured to the inside of the skin and away from the neck muscles. Tissue dehydration during the procedure was minimized with the generous application of PBS to the nerve and surrounding tissues. At the conclusion of the experiment, the animals were sacrificed and the devices recovered. Individual acute recording and stimulating experiments lasted 2–3 h in total.

### Chronic Implant Preparation (n = 3 Birds)

The bird was prepared for surgery as described above and placed in a stereotax. A sagittal incision was made along the top of the head and the tissue retracted. Four to six stainless steel anchor pins (26002‐10, Fine Science Tools) were threaded between layers of the skull, and a head cap was made from dental acrylic. The device connector was secured to the head cap with additional dental acrylic, and the μcPNI and a platinum reference wire (0.003 in dia., Teflon coated; AM‐Systems) were trocared beneath the skin to the neck. The animal was then removed from the stereotax and placed in a supine position with neck support. A 10–15 mm incision was made at the base of the neck, and the trachea and TSN were isolated as described above. The μcPNI interface was implanted on the nerve, and the reference wire was secured to the underside of the skin. Anti‐inflammatory splash block (≈250 µL 1% Meloxicam) was applied directly to the implant site, and incisions were closed with sutures. All birds exhibited normal rates of singing within 2 days of surgery.

### In Vivo Electrophysiology

All experiments were implemented and controlled using custom LabVIEW (National Instruments) and MATLAB (MathWorks) software applications. Acute electrophysiological data were recorded on the right‐side TSN using μcPNI interfaces with an RZ5 BioAmp Processor and an RA16PA Medusa Preamplifier (Tucker‐Davis Technologies). Neural signals were digitized at 24.4 kHz and 16‐bit depth and were Bessel bandpass filtered (1 Hz to 10 kHz, zero‐phase). Stimulation currents were delivered—through either bipolar silver hook electrodes or a second μcPNI—using a PlexStim programmable stimulator (Plexon). For all electrophysiology experiments, current pulses were biphasic, 200 µs phase^−1^ in duration, delivered at 1 Hz, and varied in amplitudes from −110–110 µA. By convention, positive current amplitudes were cathodic; negative amplitudes anodic. For chronic experiments, birds were recorded continuously using sound‐triggered software, generating a complete record of vocalizations and nerve activity for the experiment. Neural recordings were acquired with an RHD 2000 system with a 16‐channel unipolar input headstage (Intan Technologies), amplified, and bandpass filtered (0.3–15 kHz). Singing‐related nerve activity was recorded from six sites on the TSN in n = 3 birds.

### Data Analysis

All electrophysiology data analysis was performed off‐line using MATLAB. Activity ≈5 ms before and up to 25 ms after stimulation onset were sampled and used the onset of the stimulation artifact (Figures 5b and 7b at 0 ms) to temporally align individual trial responses. Absolute response amplitudes were observed and quantified in a stimulation response window 0.75–4 ms after stimulation onset—a latency consistent with estimated nerve conduction velocities for 4–6 µm diameter myelinated axons (i.e., 4–24 m s^−1^). An evoked response was considered to be detected if the SNR within the signal response window exceeded a 90% confidence interval calculated by bootstrap (i.e., resampling with replacement of the signal and noise intervals over n = 10 000 trials). Figures 5b and 7b show individual stimulation trials from single experimental sessions. Lines in Figure 5c,e show mean responses over n = 16–64 trials in an exemplar bird. Data points in Figures 5d and 7c show mean response at each stimulation intensity over n = 64 trials for each bird; symbols identify individual birds. Figure 5f shows the mean (solid line) and standard error (shaded region) across trials. Figure 5g shows the mean (bar) and standard deviation (error bars) across animals; symbols identify individual animals.

### Syllable Segmentation and Annotation

Raw audio recordings were segmented into syllables as previously described.^[^ ^118^ ^]^ Briefly, spectrograms were calculated for all prospective syllables, and a neural network (5000 input layer, 100 hidden layers, 3–10 output layer neurons) was trained to identify syllable types using a test data set created manually by visual inspection of song spectrograms. Accuracy of the automated annotation was verified by visual inspection of a subset of syllable spectrograms.

### Alignment of the Neural Recordings to Song

A dynamic time warping algorithm was used to align individual song motifs to a common template as previously described.^[^ ^118^ ^]^ The warping path derived from this alignment was then applied to the corresponding common mode subtracted and bandpass filtered TSN voltage recordings (0.3–6 kHz, zero‐phase, 2‐pole Butterworth) with no premotor time‐shifting. The aligned neural traces were squared (to calculate signal envelope) and smoothed (20 ms boxcar window, 1 ms advance).

### Activity Stability Correlation

The stability of recorded TSN temporal dynamics was calculated as the Pearson's correlation between the aligned neural signal envelope (averaged over 25 consecutive motifs) on the first day of recording with the same at later timepoints. The day 1 data point in Figure 6e,f denotes the correlation between the mean signal envelopes for two consecutive blocks of 25 motifs recorded on the first day. The running correlation (Figure 6e,f) shows Pearson's correlation between the mean activity envelope of 25 motifs on the first day of recording and the mean of signal envelopes in a sliding window (width: 25; advance: 1).

### Neural Activity Peak‐to‐Peak Voltage

The trial‐by‐trial peak‐to‐peak voltage of singing‐related nerve activity was calculated as the difference of the maximum and minimum voltage recorded for each song motif. The data points in Figure 6e,f denoted the mean peak‐to‐peak voltage over all trials produced in a day.

### Neural Activity Event Rate

The trial‐by‐trial event rate of singing‐related nerve activity was calculated as the number of envelope threshold crossings per unit time. A unique threshold was calculated for each motif at 5 standard deviations over the mean during singing; duration of the unwarped song was used to calculate rates. The data points in Figure 6e,f denoted the mean event rate over all trials produced in a day.

### Statistical Analysis

All statistics on data pooled across animals were reported in the main text as mean ± SD and depicted in figure error bars as mean ± SD, unless otherwise noted. Figure starring schema: *P < 0.05, **P < 0.01, and ***P < 0.001. N.S.: not significant. Where appropriate, distributions passed tests for normality (Kolmogorov‐Smirnov), equal variance (Levene), and/or sphericity (Mauchly), unless otherwise noted. Multiple comparison corrected tests were used where justified. Statistical tests for specific experiments were performed as described below:

Figure 3d: Comparison of noise RMS on L1 and L2 (n = 3 electrodes per layer). A Wilcoxon rank‐sum test showed no significant differences between RMS noise measured in L1 and L2 electrodes before (*P* = 0.4) and after (*P* = 0.7) electroplating with platinum black.
Figure 4b: Impedance as a function of bending radius for n = 3 electrodes on each layer. A one‐way ANOVA revealed no significant differences between groups (layers) at any bend radius (*F* = 0.45, *P* = 0.51).
Figure 4d: Impedance as a function of bending cycle for n = 3 electrodes on each layer. A one‐way ANOVA revealed no significant differences between groups (layers) at any cycle number (*F* = 3.68, *P* = 0.078).
Figure 4f: Impedance as a function of bending cycle for n = 2 electrodes on each layer. A one‐way ANOVA revealed no significant differences between groups (layers) at any cycle number (*F* = 3.26, *P* = 0.074).
Figure 5g: Comparison of stimulation evoked response amplitudes before and after lidocaine/saline application in n = 4 birds. A repeated‐measures ANOVA revealed significant differences between the treatments (*F* = 12.65, *P* = 0.007). Post‐hoc comparisons using Dunnett's test showed significant differences in *V*_pp_ between saline (control) and lidocaine application (*P* = 0.009); following washout, the *V*_pp_ was not significantly different from control (*P* = 0.29).
Figure 6d: Comparison of mean correlation between aligned signal envelopes recorded on the same electrode and different electrodes (n = 500 signal envelopes from each of 6 electrodes in n = 3 birds). A two‐sided, paired t‐test revealed that the correlation of envelops recorded on the same electrodes was significantly different (*P* = 0.008) from those recorded on different channels.
Figure 6f: Comparison of chronic stability metrics on the first and last day of recording in n = 3 birds. For each bird, a two‐tailed paired t‐test showed no significant differences between the 6‐channel daily mean correlations (*P* > 0.17), mean peak‐to‐peak voltages (*P* > 0.68), and event rates (*P* > 0.37) on day 1 and those on the last day.

### Code Availability

All custom code is accessible in an online repository at https://github.com/timotchy/Rowen‐et‐al‐2021.

## Conflict of Interest

The authors declare no conflict of interest.

## Supporting information

Supporting Information

## Data Availability

The data that support the findings of this study are available from the corresponding author upon reasonable request.
