# Supplementary material for: A Microclip Peripheral Nerve Interface (μcPNI) for Bioelectronic Interfacing with Small Nerves
Source: Adv Sci (Weinh). 2021 Nov 26;9(3):2102945. doi: 10.1002/advs.202102945 (PMC8787429; doi:10.1002/advs.202102945)
Supplement: Supplementary file 1 — Supporting Information [file ADVS-9-2102945-s001.pdf]

ADVANCED SCIENCE
Open Access

Supporting Information

for *Adv. Sci.*, DOI: 10.1002/advs.202102945

A Microclip Peripheral Nerve Interface (μcPNI) for Bioelectronic Interfacing with Small Nerves

*Cami C. Rowan, Oliver Graudejus, and Timothy M. Otchy**

Supporting Information

A Microclip Peripheral Nerve Interface (µcPNI) for Bioelectronic Interfacing with Small Nerves

*Cami C. Rowan, Oliver Graudejus, and Timothy M. Otchy**

Fabrication of Microelectrode Arrays: The main steps for the fabrication of the bi-layer sMEA for the µcPNI are as follows.

Step 1: PDMS Substrate. Glass slides (Wilmad LabGlass, P-3X3-0-1MTHK) were coated with a non-stick monolayer of 1 H,1 H,2 H,2 H Perfluorooctyltrichlorosilane (PFOTCS) to facilitate removal of the electrode array after fabrication. To produce the PDMS substrate, pre-polymer and crosslinker (Sylgard® 184, Dow Corning) were mixed in a 10:1 ratio by weight followed by degassing in vacuum. The mixture was spin-coated on a PFOTCS coated glass slide at 1500 rpm for 50 s, and cured for at least 12 hr at 60°C, producing the 45 µm thick elastomeric PDMS substrate.

Step 2: L1 Deposition. The first (lower) electrode layer (L1) was produced by sequential thermal evaporation (Auto 306, Edwards) of 3 nm of chromium, 35 nm of microcracked gold, and 3 nm of chromium through a custom shadow mask (Temicon GmbH, Germany). The substrate temperature did not exceed 65°C[86] and the deposition rate for the gold was at least 0.5 nm s^{-1} to produce the microcracked gold morphology.

Step 3: L1 Encapsulation. The L1 electrode layer was encapsulated by transfer bonding with a 30 µm thick layer of PDMS with contact holes for the recording sites and the contact pads on the PCB. A silicon wafer was patterned by photolithography (AZ4330 photoresist) and etching (STS ASE ICP DRIE) with the pattern of the contact holes using standard microfabrication methods, followed by coating with a non-stick layer of PFOCTS.[117] Liquid PDMS pre-polymer and crosslinker (Sylgard 184, same as substrate) were mixed in a 10:1 ratio by weight, degassed in vacuum, and poured over the patterned wafer. A 3 mm thick slab of cured PDMS (same as substrate) that was previously coated with a non-stick layer of PFOTCS[94] was placed over the uncured PDMS on the silicon wafer. Air bubbles were manually pushed out by gently pressing on the slab, and a glass slide was placed on top of the cured PDMS slab. Three 0.7" diameter neodymium magnets (07047, Master Magnetics) each were placed on the glass slide above the slab and below the silicon wafer to apply a pressure to prevent uncured PDMS from leaking into the section of the wafer that defines the contact holes. After curing for at least 12 h at 60°C, the PDMS slab with the adhering patterned encapsulation layer was peeled quickly from the wafer.[118] The surfaces of the encapsulation layer and the L1 electrodes were after plasma treated (30 W, 30 s, PlasmaEtch PE25), aligned in a custom-built aligner, and bonded. To enhance bonding, the encapsulated L1 electrodes were placed on a hot plate at 100°C for 15 min. After heat treatment, the PDMS slab was slowly peeled off, leaving behind the encapsulation layer bonded to the electrodes. It is critical that the L1 encapsulation layer is smooth without creases or wrinkles because it is the substrate for the second electrode layer (L2). This encapsulation process combines the benefits of two previously described methods while avoiding their respective disadvantages.[94,117] This process allows for efficient encapsulation of a large number of small electrodes and to produce multi-layer PNIs with high electrode density and high spatial resolution.

Steps 4 and 5: L2 Deposition and Encapsulation. The deposition and encapsulation of the second electrode layer (L2) were accomplished by repeating Steps 2 and 3, but with the shadow

mask that defines the electrodes for L2 and a patterned wafer with the contact holes for L2. It is critical that the shadow mask with the pattern of L2 is accurately aligned with the L1 traces. This was done in the custom-built tabletop aligner. Figure 1b shows details of the µcPNI array after Step 5.

Measuring µcPNI sMEA Thickness: The thickness of the µcPNI substrate, encapsulation layers, and overall thickness were measured with a microscope (AmScope). Measurements were taken after fabrication (Figure 2a) using the microscope measuring tool (Figure S1). The overall thickness of the sMEA was measured to be a total of ~105 µm. Substrate thickness was measured to be ~45 µm and encapsulation layer thickness (L1 & L2) was measured to be ~30 µm each.

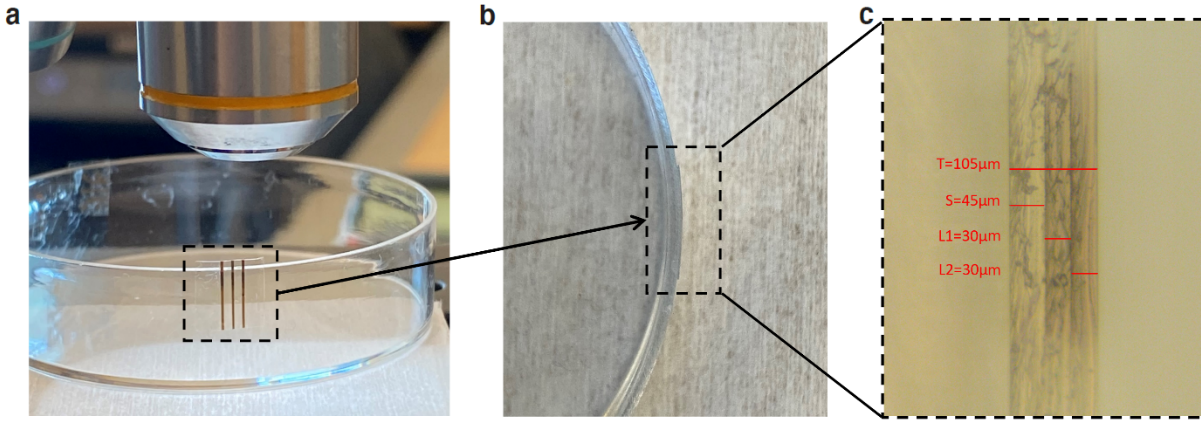

Figure S1. µcPNI sMEA thickness measured with microscope. a) The center of a µcPNI array is cut from the glass slide and placed along the outer edge of a petri dish. b) The edge of the array faces the microscope lens. c) The array thickness is measured using the microscopes measuring tool. Multiple measurements of multiple µcPNI's were averaged to a total thickness of about 105 µm (substrate 45 µm + two encapsulation layers 30 µm).

Laser cutting Microelectrode Arrays: A home-built CO_2 laser cutting system was used to pattern the PDMS electrodes before peeling them from the glass carrier. The laser had a maximum power of 50 W and maximum pulse rate of 10 MHz; a standard 50 mm objective lens generated a focal spot diameter of ~15 µm. A motorized X-Y stage (Edmunds Zaber 100x120mm) beneath the objective held the glass carrier and positioned the electrode during the cutting process. The electrode pattern was drawn in SolidWorks (Dassault Systèmes) and imported to a MATLAB interface that controlled the movement of the stage. Laser power (3 W), stage translation speed (400 mm/s), and laser pulse rates (3 MHz) were optimized to cut through the thin PDMS film and obtain patterns of ideal profile (Figure S2, right). After cutting, the PDMS was rinsed in nitromethane to eliminate ash and debris created by the CO_2 laser.

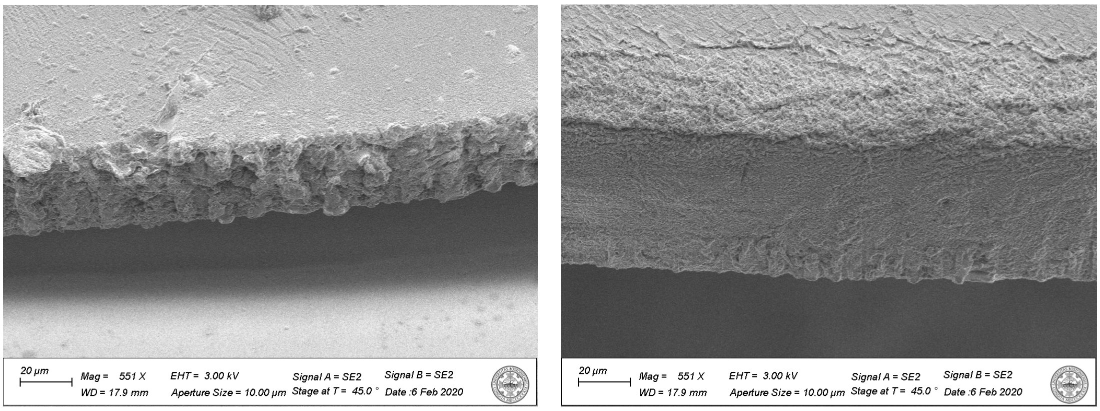

Figure S2. Edges of a laser-cut µcPNI sMEA. Laser-cut edges of µcPNI at (*left*) 4W laser power and 800m/s feed rate and (*right*) 3W laser power and 400mm/s feed rate. (Both at 3MHz pulse rate.)

Printed Circuit Board Assembly: The section of the sMEA with the contact pads was compression-bonded between two custom PCBs[85,119] with a 16-channel Omnetics connector (A79040-001, Omnetics) (Figure 1c). The Omnetics connector was soldered to the PCB with Sn-Pb solder paste (Sn63/Pb37, 247 Solder), and thermally cured following the standard solder paste reflow profile for Kester paste. Silver paste (EPO-TEK® H27D, Epoxy Technology) was used to mediate electrical contact between the contact pads on the array and the PCB. Stainless-steel screws (McMaster-Carr®) secured the two PCBs.

Tracking the electrode impedance throughout the experiment: For n = 3 devices, the impedance of each electrode was measured at different stages of the experiment to confirm the electrochemical stability of the electrode throughout the experiment. (Figure S3). All measurements were made following printing of the microclip. pre-plating, post-plating, and post-explant measurements were made with the electrode flat within the microclip; the implanted measurement was made with the electrode curled in the microclip and in contact with the tracheosyringeal nerve. The table in S3a shows the 1 kHz impedance of each electrode in each condition (reported in kΩ). Figure S3b reports these same impedances as the mean +/- standard deviation across electrodes in a device (n = 6 electrodes).

a

Device	PNI 41-1b						PNI 40-2b						PNI 52-1b					
Electrode	E1	E2	E3	E4	E5	E6	E1	E2	E3	E4	E5	E6	E1	E2	E3	E4	E5	E6
Pre-plating	594	713	178	384	427	288	376	247	295	314	855	302	697	494	659	483	507	553
Post-plating	17	14	13	29	22	24	16	26	6	11	35	13	28	21	24	18	27	19
Implanted	89	75	77	89	84	72	69	60	59	63	71	53	71	84	96	90	63	85
Post-explant	23	26	29	43	32	25	28	36	19	32	47	29	24	38	17	40	15	13

b

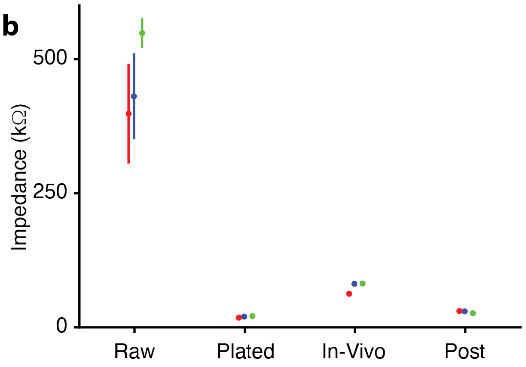

Figure S3. Electrode impedance (at 1 kHz) for µcPNIs throughout the experiments. a) electrode impedance of all six electrodes on three µcPNIs pre-plating, post-plating, while implanted and after explantation (post-explant), and b) corresponding plot showing mean and standard deviation for each device and condition.

Electroplating with Platinum Black: A potentiostat (CHI 660) was used in cyclic voltammetry mode in a 3-electrode configuration: the electrodes on the µcPNI are the working electrodes (cathode), a Pt wire is the counter electrode (anode), and a Ag/AgCl wire is the reference electrode. Prior to electroplating, the µcPNI was treated with an air plasma (30 W, 30 s, PlasmaEtch PE25), which turned the hydrophobic surface of the native PDMS hydrophilic. The plasma treated electrodes were immersed in a solution of $H_2PtCl_6 \cdot aq$ (Sigma Aldrich) and SDS (40 mM, Sigma Aldrich) in DI water.[119] Both, the plasma treatment and the addition of SDS to the electroplating solution, improve wetting of the electrode surface which reduces the likelihood of air pockets forming at the recording sites. The electrical connection between the working electrodes and the potentiostat was established with break-out cables (A79045-001, Omnetics) that attached to the Omnetics connector on the PCB (Figure S4a,b). Cyclic voltammetry was performed between -0.7 to 0.1 V at a scan rate of 50 mV s^{-1} with four sweep segments (Figure S4c). After sequentially plating all six electrodes, the µcPNI was removed from the solution, thoroughly rinsed with deionized water, and dried in air.

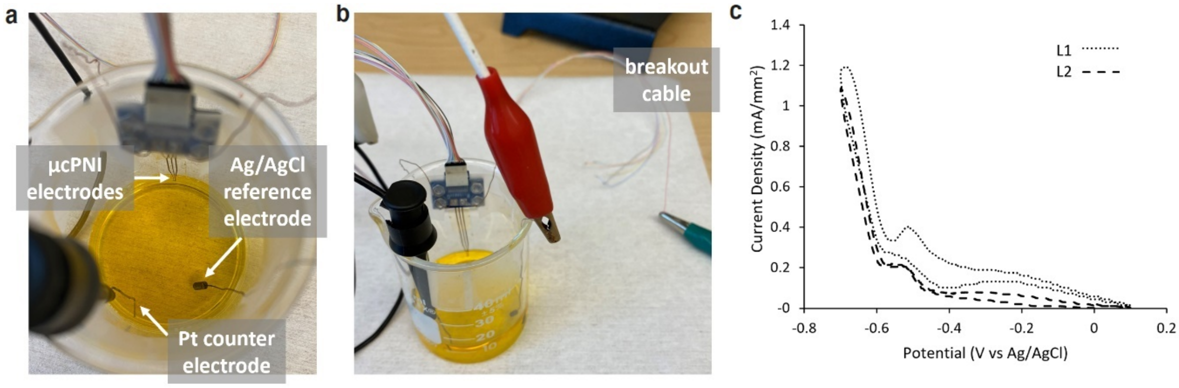

Figure S4. Experimental setup of electroplating the electrodes with platinum black. a) 3-electrode configuration (µcPNI, Pt, Ag/AgCl) in plating solution with b) breakout cable (A79045-001, Omnetics) and alligator clip connections to potentiostat (CHI 660). c) CV curve displaying current density against voltage potential for a L1 vs L2 electrode.

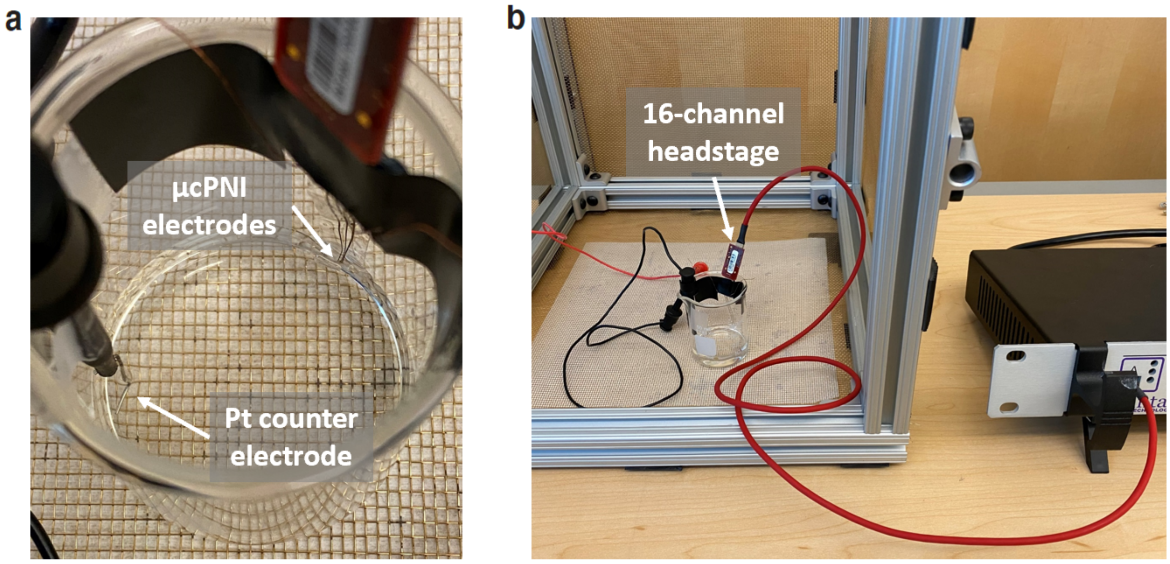

Figure S5. Setup of impedance and recording noise measurements. a) µcPNI electrodes measured against a Pt counter electrode and b) µcPNI noise measurement setup showing connection to Intan multichannel recording and stimulation system (RHS 2000).

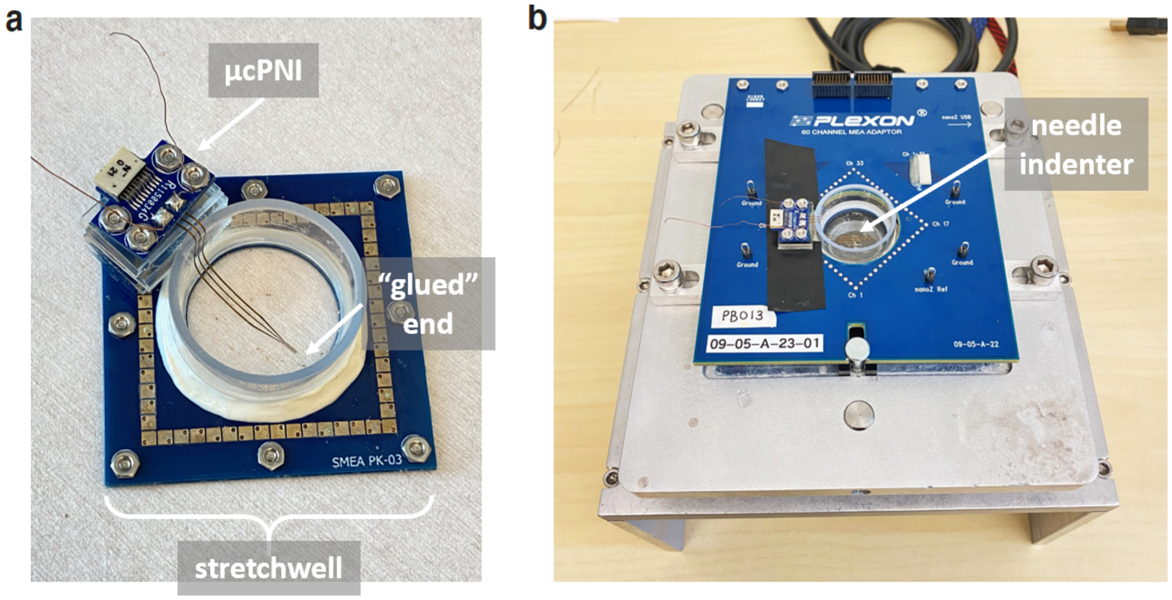

Figure S6. Experimental setup of bending fatigue testing. a) µcPNI inside PDMS stretchwell. The stretchwell's PCB is not used for electrophysiology in this study. b) µcPNI and stretchwell placed in a mechanical stretcher that fits inside an incubator. The mechanical stretcher raises and lowers the stretchwell over the needle shaft indenter, bending the µcPNI. The Plexon board is not used for electrophysiology in this study.
